# How frequency‐dependent selection affects population fitness, maladaptation and evolutionary rescue

**DOI:** 10.1111/eva.12714

**Published:** 2018-10-26

**Authors:** Erik I. Svensson, Tim Connallon

**Affiliations:** ^1^ Evolutionary Ecology Unit Department of Biology Lund University Lund Sweden; ^2^ School of Biological Sciences Monash University Clayton Victoria Australia

**Keywords:** conservation biology, costs of selection, environmental change, evolutionary rescue, frequency‐dependent selection, population extinction, quantitative genetics, sexual conflict

## Abstract

Frequency‐dependent (FD) selection is a central process maintaining genetic variation and mediating evolution of population fitness. FD selection has attracted interest from researchers in a wide range of biological subdisciplines, including evolutionary genetics, behavioural ecology and, more recently, community ecology. However, the implications of frequency dependence for applied biological problems, particularly maladaptation, biological conservation and evolutionary rescue remain underexplored. The neglect of FD selection in conservation is particularly unfortunate. Classical theory, dating back to the 1940s, demonstrated that frequency dependence can either increase or decrease population fitness. These evolutionary consequences of FD selection are relevant to modern concerns about population persistence and the capacity of evolution to alleviate extinction risks. But exactly when should we expect FD selection to increase versus decrease absolute fitness and population growth? And how much of an impact is FD selection expected to have on population persistence versus extinction in changing environments? The answers to these questions have implications for evolutionary rescue under climate change and may inform strategies for managing threatened populations. Here, we revisit the core theory of FD selection, reviewing classical single‐locus models of population genetic change and outlining short‐ and long‐run consequences of FD selection for the evolution of population fitness. We then develop a quantitative genetic model of evolutionary rescue in a deteriorating environment, with population persistence hinging upon the evolution of a quantitative trait subject to both frequency‐dependent and frequency‐independent natural selection. We discuss the empirical literature pertinent to this theory, which supports key assumptions of our model. We show that FD selection can promote population persistence when it aligns with the direction of frequency‐independent selection imposed by abiotic environmental conditions. However, under most scenarios of environmental change, FD selection limits a population's evolutionary responsiveness to changing conditions and narrows the rate of environmental change that is evolutionarily tolerable.

## 1 INTRODUCTION

Frequency‐dependent (FD) selection—in which the fitness of a genotype or phenotype depends on its frequency within the population (Lande, 1976)—is an important and widely recognized process in evolutionary biology, first described by early mathematical population geneticists in the field, particularly Sewall Wright and Ronald Fisher (Fisher, 1930; Svensson, 2018; Wright, 1969). For example, Fisher's theory for the evolution of equal sex ratios begins with the intuition that parents producing the minority sex should have a fitness advantage over those that invest in the majority sex; the equal genetic contributions of mothers and fathers to offspring necessarily lead to a negative frequency‐dependent advantage of producing members of the rarer sex (Fisher, 1930). Fisher was also first to note that sexual selection by female choice had the power to generate positive frequency‐dependent feedback between female preferences and extravagant male traits, leading to “runaway” evolutionary change of male traits *and* female preferences (Fisher, 1930; Kirkpatrick, 1982; Lande, 1981; Mead & Arnold, 2004; Prum, 2010; Svensson, 2018). Sewall Wright, who wrote extensively about frequency‐dependent selection (Wright, 1969), was the first to note that natural selection need not maximize mean fitness of a population. Rather, FD selection can, in some cases, drive evolutionary reductions in population fitness (Wright, 1942). This insight of Wright's is sometimes overlooked, given his tendency to emphasize fitness maximization in other contexts of natural selection (reviewed in Li, 1955; Grodwohl, 2017).

In the 1960s and 1970s, FD selection became popular among behavioural ecologists who were interested in animal conflict and cooperation, culminating in the development of evolutionary game theory (Maynard Smith, 1979, 1982) and, subsequently, adaptive dynamics (Waxman & Gavrilets, 2005). The phenotype‐oriented modelling tools of game theory and adaptive dynamics promoted theoretical exploration of a broad range of interactions between individuals, some of which generated complex forms of FD selection. Game theory and adaptive dynamics substantially expanded upon the foundational models of population genetics, yet the assumptions and mathematical restrictions of this branch of theory sometimes came at a cost, as many questions in evolutionary biology—particularly those pertaining to short‐term evolutionary change, the evolution of population fitness and the maintenance of genetic variation—are less tractable within the adaptive dynamics framework than they are within the classical modelling traditions in evolutionary genetics (Lion, 2018; Maynard Smith, 1982; Spencer & Feldman, 2005; Wright, 1969). We expand on these points further below.

In contrast to the situation in the fields of behavioural ecology and social evolution, evolutionary genetics theory from the 1990s and onward became increasingly focused on models of mean absolute fitness, particularly as they pertain to population dynamics in changing environments (Bell, 2017; Gomulkiewicz & Holt, 1995; Gomulkiewicz & Houle, 2009; Lynch & Lande, 1993; Orr & Unckless, 2014). Yet this branch of theory primarily focused on frequency‐*independent* forms of selection and largely neglected frequency‐dependent processes that are common to many animal populations (Ayala & Campbell, 1974; Sinervo & Calsbeek, 2006). For instance, frequency‐dependent selection has been empirically demonstrated to be important in maintaining sexually selected colour polymorphisms within populations (Sinervo & Lively, 1996; Wellenreuther, Svensson, & Hansson, 2014) and it can also affect short‐ and long‐term predictability of evolutionary dynamics on ecological time scales (Nosil et al., 2018; Svensson, Abbott, & Hardling, 2005) and can constrain population divergence by favouring rare immigrant phenotypes (Bolnick & Stutz, 2017). Frequency‐dependent processes such as rare‐species advantage can also operate in ecological communities among species, and such processes can maintain local diversity (Harpole & Suding, 2007; Svensson, Gómez‐Llano, Torres, & Bensch, 2018; Wills et al., 2006).

The last decade has witnessed a pronounced growth in evolutionary models that explicitly link genetics, selection and population dynamics, to characterize the demographic costs of maladaptation to climate change and the capacity of evolution to resolve these costs (Bell, 2017). For the purpose of this theme issue, we define maladaptation as the deviation of a population from its adaptive peak (Crespi, 2000). This growing body of theory is central to predictions about “evolutionary rescue” and the potential for evolution to maintain high fitness in the face of environmental deterioration (Bell, 2017; Chevin, Lande, & Mace, 2010). In such contexts, the maintenance of high absolute fitness is critical. A viable population must maintain high mean fitness among its members, despite perpetual change in the environment (Chevin et al., 2010; Lynch & Lande, 1993). Well‐adapted populations are able to sustain large and stable population sizes, whereas maladapted ones exhibit sub‐replacement fertility that ultimately leads to extinction if left uncorrected by adaptive evolutionary change.

The relationship between population mean fitness, natural selection and the genetic composition of populations is difficult to predict when selection is frequency‐dependent, which has historically been a source of controversy underlying debates about the utility of the adaptive landscape metaphor and the question if selection maximizes population fitness (Fear & Price, 1998; Grodwohl, 2017; Kaplan, 2008; Okasha, 2018; Pigliucci, 2008; Rice, 2004; Svensson, 2016; Svensson & Calsbeek, 2012). Here, we develop a quantitative genetic model that is inspired by adaptive landscape theory and the existence of adaptive peaks, which has empirically turned out to be a useful and successful approach (Arnold, Pfrender, & Jones, 2001; Chenoweth, Hunt, & Rundle, 2012; Svensson & Calsbeek, 2012), although we are aware of the criticisms from some philosophers and theoretical population geneticists who have questioned the idea of fitness maximization behind adaptive landscapes (Grodwohl, 2017; Kaplan, 2008; Moran, 1963; Okasha, 2018; Pigliucci, 2008). We ask the question: To what extent does frequency‐dependent selection mediate increased or decreased population mean fitness, particularly under changing conditions? This question is not only of interest for our basic understanding of evolutionary processes, but also has important applied consequences for extinction (Chevin, Gallet, Gomulkiewicz, Holt, & Fellous, 2013; Gomulkiewicz & Holt, 1995) and agricultural productivity (Weiner, Andersen, Wille, Griepentrog, & Olsen, 2010), which are intimately linked to population mean fitness. Prior theory suggests a range of potential outcomes of FD selection for population persistence, but provides no straightforward answer to the question. For example, classical population and quantitative genetics theory (Lande, 1976; Wright, 1942) clearly shows that that FD selection, acting on discrete phenotypes or on continuous traits, can cause mean population fitness to increase *or* to decrease, at least during short evolutionary intervals (Box 1) (Fear & Price, 1998; Rice, 2004; Svensson, 2016). In this sense, FD selection is one of several forms of adaptive evolution that can, in principle, reduce population fitness (Grodwohl, 2017; Leigh, 1977; Moran, 1963; Rankin, Bargum, & Kokko, 2007). Yet, which of the two potential consequences of FD selection is most likely to occur in nature—an increase or a decrease in population fitness? Are negative effects of FD selection likely to arise in contexts of environmental change? If so, what is the quantitative impact of these negative effects on the potential for evolutionary rescue?

Box 1 **Frequency‐dependent selection and short‐term changes to mean fitness**

1 Much of the early theory of frequency‐dependent selection and its impact on population fitness is attributable to Sewall Wright, who recognized that selection can decrease mean fitness when genotypic fitnesses are frequency‐dependent (Heino, Metz, & Kaitala, 1998; Li, 1955; Wright, 1942, 1949, 1969). The basis of this theoretical conclusion can be illustrated with a simple model of a single, haploid locus with two genotypes, *A* and *B*, at population frequencies of *p* and *q *=* *1 – *p*, respectively. Individuals that carry the *A* genotype have a fitness of *W*_*A*_, and individuals carrying the *B* genotype have a fitness of *W*_*B*_, both of which can be functions of *p*. Mean population fitness is:

(1.1) $$\bar{W}=pW_{A}+\left(1-p\right)W_{B}$$

**Short‐term changes to mean fitness**

In a continuous‐time, continuous‐population evolutionary model, the rate of change of mean fitness is:

(1.2) $$\frac{d\bar{W}}{dt}=\frac{d\bar{W}}{dp}\cdot \frac{dp}{dt},$$

where the $d\bar{W}/dp$ represents change in mean fitness with change in the frequency of genotype *A*, and d*p*/d*t* is the rate of change of the frequency of *A*. The elements of equation (1.2) are:

(1.3a) $$\frac{d\bar{W}}{dp}=W_{A}-W_{B}+p\frac{dW_{A}}{dp}+\left(1-p\right)\frac{dW_{B}}{dp}=W_{A}-W_{B}+E\left[\frac{dW}{dp}\right],$$

and

(1.3b) $$\frac{dp}{dt}=p\left(1-p\right)\left(W_{A}-W_{B}\right)=p\left(1-p\right)\left(\frac{d\bar{W}}{dp}-E\left[\frac{dW}{dp}\right]\right),$$

where E[d*W*/d*p*] = *p*(d*W*_*A*_/d*p*) + (1 – *p*)(d*W*_*B*_/d*p*) represents the average change in genotype fitness with change in allele frequency—a measure of the pattern of frequency dependence.

Substituting Equations (1.3a, 1.3b) into Equation (1.2) provides an expression for the change in mean fitness is over a single generation:

(1.4) $$\frac{d\bar{W}}{dt}=V_{A}+E\left(\delta W\right)$$

(which follows the notation of a discrete‐time version of the model by Rice, 2004). The first term of Equation (1.4) represents the additive genetic variance for fitness, $V_{A}=p\left(1-p\right){\left(W_{A}-W_{B}\right)}^{2}$, which is positive or zero. The second term, $E\left(\delta W\right)=\frac{dp}{dt}\cdot E\left[\frac{dW}{dp}\right]$, describes the “average change in genotypic fitness due to the effects of frequency‐dependence” (Rice, 2004, p. 35); $E\left(\delta W\right)$ can be positive or negative, depending on the pattern of frequency dependence of fitness for the two genotypes. If positive, it contributes to a net increase in fitness over time. If negative, it can either dampen the net increase in fitness (when $V_{A}>-E\left(\delta W\right)$), or cause fitness to decline (when $V_{A}<-E\left(\delta W\right)$).

**An example of frequency‐dependent selection decreasing mean fitness**

Suppose that *W*_*A*_ = *V* – *pa*, and *W*_*B*_ = *V*(1 – *s*), where *V*,* s*, and *a* are positive constants with the constraint: *V *> *a *> *Vs*. In this case, the relative fitnesses of the two genotypes are negative frequency‐dependent. Each is favoured when rare, and there is a stable polymorphic equilibrium, with the *A* genotype at a frequency of $\hat{p}=Vs/a$, and equilibrium mean fitness $\bar{W}=V\left(1-s\right)$; the stable polymorphic equilibrium is reached when *W*_*A*_ = *W*_*B*_. However, mean fitness is maximized at the non‐equilibrium frequency: $p=\hat{p}/2$. Mean fitness declines when the frequency of A is within the range: $\hat{p}/2<p<\hat{p}$.

**Relation to Fisher's fundamental theorem of natural selection**

Fisher discussed how two general factors affect change in mean population fitness over time. First, mean fitness can increase as a result of evolution by natural selection. In his Fundamental Theory of Natural Selection (Fisher, 1930, pp. 34–35), Fisher showed that the rate of change in mean fitness, holding the environment constant, is proportional to the additive genetic variance for fitness, as represented by *V*_*A*_ in Equation (1.4). Second, changes in the environment affect the way in which fitness is expressed; environmental change should tend to decrease fitness because populations are, at best, only adapted to environmental conditions that occurred in the past—a so‐called lag load (Chevin, 2013). Continual change in the environment—through changes in abiotic conditions and the nature of interactions between individuals from the same or from different species—causes “deterioration of the environment” with respect to fitness (Fisher, 1930, pp. 41–42). Thus, frequency dependence, which may lead to a decline of mean fitness by way of the second term of Equation (1.4) (i.e., *E*(δ*W*)), is just one of many possible forms of environmental change that can lead to maladaptation (see Frank & Slatkin, 1992).

To establish baseline expectations for the possible effects of FD selection on the evolution of mean fitness, we first revisit classical population genetics theory for FD selection. We capture the essence of the classical theory in a haploid version of Wright's (1942) single‐locus model of FD selection, which predicts change in mean fitness over a single generation, similar to Fisher's Fundamental Theorem of Natural Selection (see Box 1). To explore longer‐run dynamics of mean fitness under the classical theory, we explore Smouse's (1976) model of evolution under frequency‐ *and* density‐dependent selection (Box 2). These classical population genetic models demonstrate how FD selection can drive short‐term decreases in mean fitness (Box 1), though fitness reductions need not persist indefinitely, as demographically stable and density‐regulated populations eventually evolve replacement fitness (i.e., no net change in population size), despite FD selection (Box 2).

Box 2 **Frequency dependence, density dependence and long‐term population size**

1 Classical population genetics theory shows that natural selection can, in principle, lead to short‐term increases or decreases in mean fitness (Box 1). On the other hand, in demographically stable populations mean absolute fitness will ultimately be dominated by density‐dependent effects on population dynamics (Heino et al., 1998). To explore the long‐run evolutionary consequences of frequency‐dependent selection for mean fitness and population size, we need a model that explicitly tracks population dynamics during evolution.

Smouse (1976) presented perhaps the simplest population genetic model that captures the joint effects of frequency‐ and density‐dependent selection during evolution. Adopting his framework, we can track the dynamics of evolution and population size for a haploid population with two genotypes (*A* and *B*). Evolutionary dynamics depend on the population growth rates for each of the two genotypes, as described by a pair of differential equations:

(2.1a) $$\frac{dN_{A}}{dt}=N_{A}\left(r_{A}-\beta_{\mathrm{AA}}N_{A}-\beta_{\mathrm{AB}}N_{B}\right),$$

and

(2.1b) $$\frac{dN_{B}}{dt}=N_{B}\left(r_{B}-\beta_{\mathrm{BA}}N_{A}-\beta_{\mathrm{BB}}N_{B}\right),$$

where *r*_*i*_ is the intrinsic growth rate for the *i*th genotype (*i *= {*A*,* B*}), *N*_*i*_ is the number of individuals carrying the *i*th genotype, *β*_*ii*_ depicts the negative density‐dependent effects of individuals of genotype *i* on other individuals of the same genotype, and *β*_*ij*_ depicts the negative density‐dependent effects of individuals of genotype *j* on individuals of genotype *i* (with negative density dependence, *β*_*ii*_, *β*_*ij*_ > 0). Note that parameters *β* and *r* can be directly related to the concept of “carrying capacity” (*K*) in models of density‐dependent population growth, where, for a population with a single genotype, *K *= *r*/*β* (Smouse, 1976).

The contribution of a genotype to population growth provides a measure of the absolute fitness of the genotype (Crow & Kimura, 1970, pp. 190‐192). We can, therefore, define fitness for genotype *A* as:

(2.2a) $$W_{A}=r_{A}-N\left(\beta_{\mathrm{AA}}p+\beta_{\mathrm{AB}}\left(1-p\right)\right)$$

where *p *= *N*_*A*_/(*N*_*A*_ + *N*_*B*_) is the frequency of genotype *A*, and *N *= *N*_*A*_ + *N*_*B*_ is the total size of the population. The fitness of genotype *B* is:

(2.2b) $$W_{B}=r_{B}-N\left(\beta_{\mathrm{BA}}p+\beta_{\mathrm{BB}}\left(1-p\right)\right)$$

Both expressions are density‐ and frequency‐dependent. Mean fitness is $\bar{W}=W_{A}p+W_{B}\left(1-p\right)$. Analysis of eqs. (2.1‐2.2) shows that the evolutionary dynamics of genotype *A* conform to the classical population genetic framework of frequency‐dependent selection (Equation (1.3b)), from Box 1:

(2.3) $$\frac{dp}{dt}=\frac{d}{dt}\left(\frac{N_{A}}{N_{A}+N_{B}}\right)=p\left(1-p\right)\left(W_{A}-W_{B}\right)=p\left(1-p\right)\left[\frac{d\bar{W}}{dp}-E\left(\frac{\mathrm{dW}}{dp}\right)\right]$$

where $E\left(\frac{dW}{dp}\right)=p\frac{dW_{A}}{dp}+\left(1-p\right)\frac{dW_{B}}{\mathrm{dp}}$.

**Equilibrium fitness and population size**

At equilibrium, and assuming the population has not gone extinct, there are three possible equilibrium states: (a) *N*_A_ = 0 and *N*_B_ = *r*_B_/*β*_BB_; (b) *N*_B_ = 0 and *N*_A_ = *r*_A_/*β*_AA_; and (c) $N_{A}=\frac{r_{A}\beta_{\mathrm{BB}}-r_{B}\beta_{\mathrm{AB}}}{\beta_{\mathrm{AA}}\beta_{\mathrm{BB}}-\beta_{\mathrm{AB}}\beta_{\mathrm{BA}}}$ and $N_{B}=\frac{r_{B}\beta_{\mathrm{AA}}-r_{A}\beta_{\mathrm{BA}}}{\beta_{\mathrm{AA}}\beta_{\mathrm{BB}}-\beta_{\mathrm{AB}}\beta_{\mathrm{BA}}}$. The condition for maintaining genetic polymorphism (equilibrium 3) is $\frac{\beta_{\mathrm{AA}}}{\beta_{\mathrm{BA}}}>\frac{r_{A}}{r_{B}}>\frac{\beta_{\mathrm{AB}}}{\beta_{\mathrm{BB}}}$, where *β*_*AA*_ *β*_*BB*_ − *β*_*AB*_ *β*_*BA*_ > 0 (see Smouse, 1976). In all three cases, it can be shown that mean fitness always reduces to $\bar{W}=0$, which corresponds to no net population growth in the continuous‐time model. Thus, while frequency‐dependent selection can induce short‐term declines in mean fitness, such declines need not persist indefinitely if density‐dependent factors ultimately dominate in the long‐run.

On the other hand, the long‐run sustainable size of a population could provide an alternative measure of a population's fitness, as argued in some empirical studies of frequency‐dependent selection (Takahashi, Tanaka, Yamamoto, Noriyuki, & Kawata, 2018). Returning to Smouse's model, we can evaluate how the equilibrium population size depends on the density‐dependent interactions between individuals with the same and those with different genotypes. Genetic polymorphism is sometimes maintained when the fitness of each genotype is negative frequency‐dependent—a scenario that occurs when the strength of competition for resources is more intense between individuals with the same genotype than between individuals with different genotypes (i.e., *β*_*AA*_ and *β*_*BB*_ are sufficiently large relative to *β*_*AB*_ and *β*_*BA*_). In a polymorphic population, the equilibrium population size will be:

(2.4) $$N=\frac{r_{A}\beta_{\mathrm{BB}}-r_{B}\beta_{\mathrm{AB}}+r_{B}\beta_{\mathrm{AA}}-r_{A}\beta_{\mathrm{BA}}}{\beta_{\mathrm{AA}}\beta_{\mathrm{BB}}-\beta_{\mathrm{AB}}\beta_{\mathrm{BA}}}$$

which exceeds the size of an equilibrium population that lacks genetic variation. This last point becomes obvious if we consider a symmetric version of the model, where *r *= *r*_*A*_ = *r*_*B*_, *β*_*ij*_ = *β*_*AB*_ = *β*_*BA*_, *β*_*ii*_ = *β*_*AA*_ = *β*_*BB*_, so that the equilibrium population size becomes:

(2.5) $$N=\frac{2r}{\beta_{\mathrm{ii}}\left(1+\beta_{\mathrm{ij}}/\beta_{\mathrm{ii}}\right)}$$

Equation (2.5) shows that population size increases as the intensity of between‐genotype competition decreases (*β*_*ij*_/*β*_*ii*_ decreases). When individuals with different genotypes utilize completely different resources (so that *β*_*ij*_/*β*_*ii*_ = 0), then a polymorphic population will grow to be twice as large as a population where either of the two alleles is fixed.

To incorporate FD selection into the broader theory of evolutionary rescue, we develop and analyse a simple model of evolutionary rescue in a population experiencing both FD selection and directional change in the abiotic environment. By modelling evolution of the intrinsic growth rate of the population—which determines whether it will persist or decline to extinction—we show that FD selection can substantially impact a population's ability to track a shifting environmental optimum and remain demographically viable. Although FD selection can sometimes rescue an otherwise doomed population, our model predicts that frequency dependence should generally hinder population persistence—perhaps substantially. We close by reviewing the empirical literature of FD selection in light of the theory. We argue that the frequency‐dependent processes outlined in our model are likely to apply broadly to many animal populations. These processes may therefore be important in determining the fates of threatened populations.

## 2 ENVIRONMENTAL CHANGE, FREQUENCY‐DEPENDENT SELECTION AND EVOLUTIONARY RESCUE

Frequency‐dependent selection represents one of many factors that can cause maladaptation. For example, in Fisher's general concept of “environmental deterioration” (Box 1), both frequency dependence and changes in the external environment can contribute to reductions of population fitness that must be compensated by evolutionary adaptation if the population is to persist. The capacity of evolution to offset environmental change, and maintain adaptation and a stable population size, is often referred to as evolutionary rescue (Bell, 2017; Stewart et al., 2017). Under evolutionary rescue, whether a population persists or goes extinct depends on the outcome of a race between adaptive evolution, which typically increases fitness and population growth, and environmental change, which leads to maladaptation and elevates extinction risk (Bell, 2017; Orr & Unckless, 2014).

Whether frequency‐dependent selection plays a role in extinction has received considerable attention in adaptive dynamics models (Waxman & Gavrilets, 2005), which have identified several hypothetical scenarios where FD selection can drive populations extinct, including in stable environments (i.e., “Darwinian extinction”, “evolutionary suicide” or “Tragedy of the Commons”; see Webb, 2003; Parvinen, 2005; Rankin, Dieckmann, & Kokko, 2011). However, key features of the adaptive dynamics tradition place it outside the arena of most evolutionary rescue theory (for exceptions, see Ferriere & Legendre, 2013; Osmond & de Mazancourt, 2013). More specifically, adaptive dynamics models are constructed on the assumption that evolutionary processes are slow relative to ecological dynamics and that evolution is slow and mutation‐limited rather than fast and acting on standing genetic variation (Barrett & Schluter, 2008; Lion, 2018). Adaptive dynamics models therefore typically focus on long‐run evolutionary states of phenotypes rather than non‐equilibrium dynamics, in which the genetic basis of phenotypic evolution is critical (Waxman & Gavrilets, 2005). These features of adaptive dynamics make the approach useful to model long‐term evolution of traits that mediate complex interactions between individuals in a population and find evolutionary endpoints. However, adaptive dynamics is not naturally suited for modelling evolutionary rescue, where evolution typically proceeds faster and on ecological timescales (Hendry, 2016; Lion, 2018; Svensson & Gosden, 2007), and where the genetic basis of fitness‐mediating traits is central to the rate of evolution and the probability of persistence. It should be noted that there have been recent attempts to better integrate quantitative genetics and adaptive dynamics models through the common theme of environmental feedbacks (Lion, 2018).

Most models of evolutionary rescue are grounded within the classical theoretical traditions of population and quantitative genetics. This modelling approach allows for non‐equilibrium evolutionary dynamics and rapid adaptation of traits affecting population growth (i.e., adaptation on ecological timescales), providing a direct and explicit link between genetic details and evolutionary outcomes **(**Bell, 2017; Bell & Collins, 2008; Chevin et al., 2010; Kopp & Matuszewski, 2014; Lande & Shannon, 1996; Lynch & Lande, 1993; Orr & Unckless, 2008, 2014). On the other hand, most evolutionary rescue models focus on frequency‐independent selection driven by changing abiotic conditions (but see Yamamichi & Miner, 2015; Osmond, Otto, & Klausmeier, 2017). Classical evolutionary theory raises the spectre of extinction due to frequency‐dependent selection (see Lande, 1976), yet such models are generally not considered in evolutionary rescue scenarios.

### 2.1 Asymmetric frequency‐dependent selection and adaptation to a changing environment

**Figure 1:**
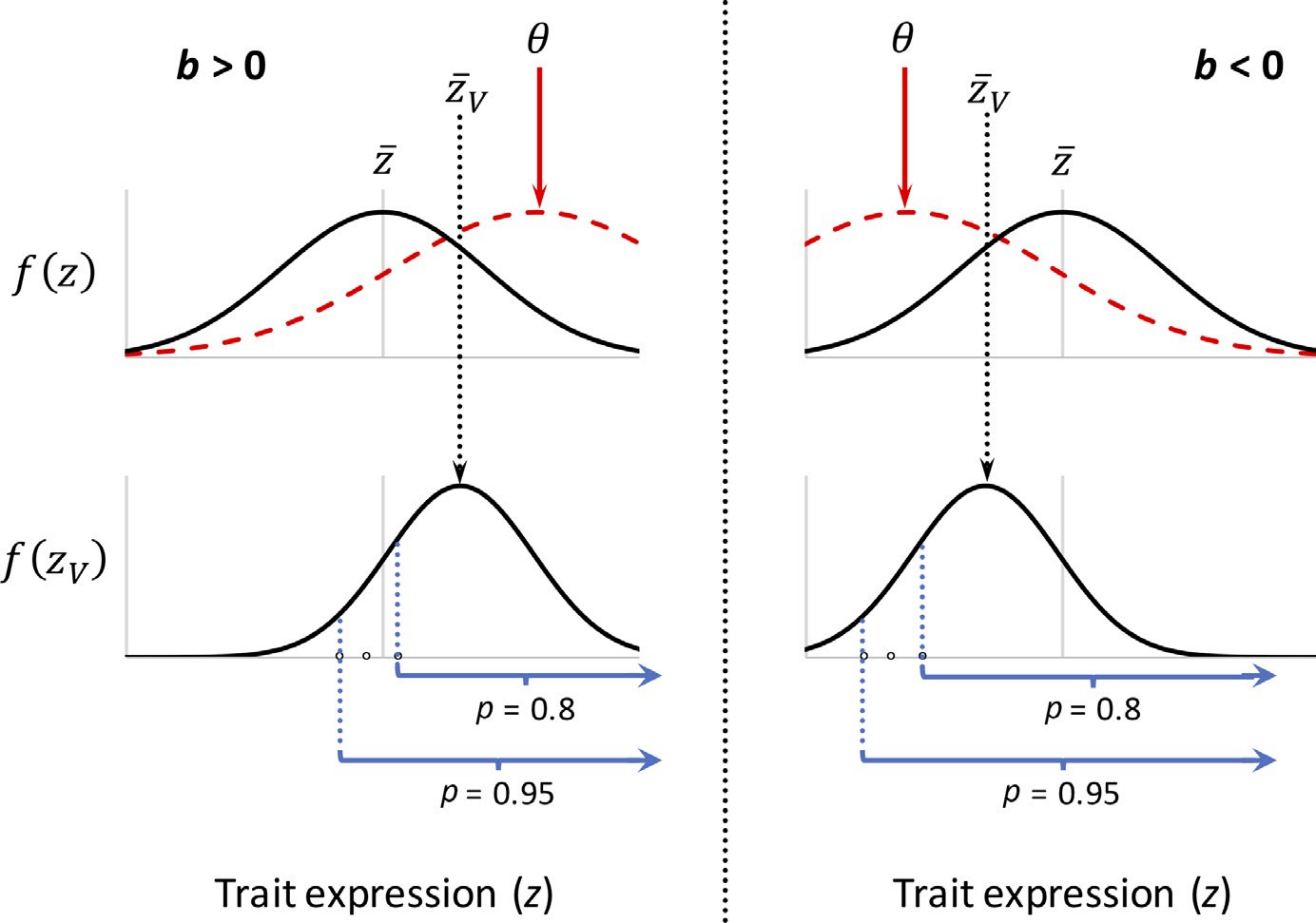
Directional selection through two fitness components. The first component of selection is frequency‐independent towards an environmental optimum (top), and the second is frequency‐dependent in favour of individuals with larger traits (bottom). In the *left‐hand panel*, FD selection is aligned with the direction of change in the environmental optimum (*b *>* *0, where *b* defines the rate and direction of change of the environmental optimum). As such, FD selection can promote adaptation and population persistence. In the *right‐hand panel*, FD selection is misaligned with the direction of change in the environmental optimum (*b *<* *0) and therefore hinders adaptation and population persistence. For additional details, see Box 3 and the main text. Prior to either episode of selection, the probability density function for the trait is *f*(*z*), with a population mean of $\overset{¯}{z}$. Following frequency‐independent selection but prior to frequency‐independent selection, the probability density function is *f*(*z*_*V*_), with a trait mean of ${\overset{¯}{z}}_{v}$. θ denotes the environmental optimum, and 1 – *P* represents the fraction of adults (those that survived frequency‐independent selection) that are selectively removed from the breeding population

To explore the consequences of frequency‐dependent selection for evolutionary rescue, we developed a simple model of population persistence that depends on the evolution of a quantitative trait affecting two major fitness components (Figure 1; Box 3; for a related model, see Lande, 1976; pp. 317–319). In our model, survival during early life is affected by abiotic environmental factors (e.g., climatic conditions); for this first fitness component, the trait is subject to *frequency‐independent stabilizing selection* to an environmentally determined optimum that shifts over time. The changing environment generates directional selection to track the moving optimum—a pattern of environment‐mediated selection that has been considered in several influential models of evolutionary rescue (see Lynch & Lande, 1993; Chevin et al., 2010; Bell, 2017). In these previous models, conditions for persistence are defined by population‐specific features genetic variation, demography and life‐history (Chevin et al., 2010; Hoffmann & Sgro, 2011; see below).

Box 3 **A quantitative genetic model of frequency‐dependent selection and evolutionary rescue**

1 In our quantitative genetic model of evolutionary rescue, each generation goes through the following life cycle: (a) birth, (b) frequency‐independent viability selection, (c) frequency‐dependent selection, where individuals from the lower portion of the trait distribution are removed from the breeding population (e.g., asymmetric frequency‐dependent selection on survival or reproductive success), and (d) reproduction and death.

**Calculating the selection differential**

At birth, the focal trait is normally distributed with mean $\overset{¯}{z}$ and phenotypic variance *σ* ^2^. Under frequency‐independent viability selection, the survival probability of an individual expressing trait value *z* is:

(3.1) $$w\left(z\right)=w_{\max}\exp\left(-\frac{{\left(\theta -z\right)}^{2}}{2\omega^{2}}\right),$$

where *w*_max_ is the survival probability of an individual expressing a trait matching the environmental optimum, *θ*, and *ω* ^2^ defines the rate of decline in survival with distance from the optimum (*w*_max_, *ω* ^2^ > 0). Mean early survival is:

(3.2) $${\bar{W}}_{V}=\int_{-\infty }^{\infty }w\left(z\right)f\left(z\right)dz=\frac{w_{\max}\sqrt{\omega^{2}}}{\sqrt{\omega^{2}+\sigma^{2}}}\exp\left(-\frac{{\left(\theta -\overset{¯}{z}\right)}^{2}}{2\left(\omega^{2}+\sigma^{2}\right)}\right),$$

where *f*(*z*) is the probability density function of the trait. Following viability selection (denoted with “*V*” subscripts), the distribution of the trait remains normal with mean and variance of ${\overset{¯}{z}}_{V}=\left(\theta \sigma^{2}+\overset{¯}{z}\omega^{2}\right)/\left(\omega^{2}+\sigma^{2}\right)$ and $\sigma_{V}^{2}=\sigma^{2}\omega^{2}/\left(\omega^{2}+\sigma^{2}\right)$. The selection differential by way of viability selection is $S_{V}={\overset{¯}{z}}_{V}-\overset{¯}{z}=\sigma^{2}\left(\theta -\overset{¯}{z}\right)/\left(\omega^{2}+\sigma^{2}\right)$.

Following viability selection, the proportion of adults that eventually breed, *P*, is defined as:

(3.3) $$P=\int_{\alpha }^{\infty }\frac{1}{\sqrt{2\pi \sigma_{V}^{2}}}\exp\left(-\frac{{\left({\overset{¯}{z}}_{V}-z\right)}^{2}}{2\sigma_{V}^{2}}\right)dz=\frac{1}{2}\left[1+\text{erf}\left(\frac{{\overset{¯}{z}}_{V}-\alpha }{\sqrt{2\sigma_{V}^{2}}}\right)\right],$$

where erf(.) is the error function, and *α* is the frequency‐dependent selection cut‐off (the truncation point of selection). The latter is defined as $\alpha ={\overset{¯}{z}}_{V}-\sqrt{2\sigma_{V}^{2}}{\text{erf}}^{-1}\left(2P-1\right)$, where erf^−1^(.) is the inverse error function. The mean trait value among breeding individuals (following frequency‐dependent selection) is:

(3.4) $${\overset{¯}{z}}_{\mathrm{FD}}=\frac{1}{P}\int_{\alpha }^{\infty }z\frac{1}{\sqrt{2\pi \sigma_{V}^{2}}}\exp\left(-\frac{{\left({\overset{¯}{z}}_{V}-z\right)}^{2}}{2\sigma_{V}^{2}}\right)dz={\overset{¯}{z}}_{V}+\frac{1}{P}\sqrt{\frac{\sigma_{V}^{2}}{2\pi }}e^{-{\left[{\mathrm{erf}}^{-1}\left(2P-1\right)\right]}^{2}}$$

which is equivalent to previous results for truncation selection (see Crow & Kimura, 1970, pp. 225–230). The selection differential across both episodes of selection is:

(3.5) $$S={\overset{¯}{z}}_{\mathrm{FD}}-\overset{¯}{z}=\frac{\sigma^{2}\left(\theta -\overset{¯}{z}\right)}{\omega^{2}+\sigma^{2}}+\frac{\sqrt{\sigma^{2}\omega^{2}}}{P\sqrt{2\pi \left(\omega^{2}+\sigma^{2}\right)}}e^{-{\left[{\mathrm{erf}}^{-1}\left(2P-1\right)\right]}^{2}},$$

Following standard quantitative genetic theory, the response to selection over one generation is *h* ^2^ *S*, where *h* ^2^ is the trait's heritability.

**Evolution and population dynamics**

We assume that the environmental optimum changes at a rate of *b*, per generation, whereas the trait's heritability (*h* ^2^), variance (*σ* ^2^) and remaining fitness landscape parameters (*P*,* W*_max_, *ω* ^2^) are constant across generations. Under these assumptions, the population eventually reaches a steady‐state where the displacement of the trait mean at birth from the environmental optimum is:

(3.6) $${\left(\theta -\overset{¯}{z}\right)}_{eq.}=\frac{b\left(\omega^{2}+\sigma^{2}\right)}{h^{2}\sigma^{2}}-\frac{\sqrt{\omega^{2}\left(\omega^{2}+\sigma^{2}\right)}}{P\sqrt{2\pi \sigma^{2}}}e^{-{\left[{\mathrm{erf}}^{-1}\left(2P-1\right)\right]}^{2}},$$

which is obtained by equating *h* ^2^ *S* and *b*. In the absence of density‐dependent effects, mean absolute fitness is $\bar{W}={\bar{W}}_{V}PR$, where *R* is the mean number of offspring produced by breeding adults (i.e., following frequency‐dependent selection). At steady state, mean fitness is:

(3.7) $$\bar{W}=PR\frac{w_{\max}\sqrt{\omega^{2}}}{\sqrt{\omega^{2}+\sigma^{2}}}\exp\left(-\frac{{\left(\theta -\overset{¯}{z}\right)}_{eq.}^{2}}{2\left(\omega^{2}+\sigma^{2}\right)}\right)$$

At low population density, the change in population size over a single generation is described by $\Delta N=\left(\bar{W}-1\right)N$ (Chevin et al., 2010). A positive intrinsic growth rate of the population requires that $\bar{W}>1$ (or $ln(\bar{W})>0$). If the steady‐state intrinsic growth rate is positive, then the population will persist, and otherwise it will go extinct. The population can persist when the following condition holds:

(3.8) $${\left(\theta -\overset{¯}{z}\right)}_{eq.}^{2}<\frac{2}{\gamma }\left(r_{\max}+\ln\left[P\right]\right)$$

where *γ* = 1/(*σ* ^2^ + *ω* ^2^) is the strength of stabilizing selection and $r_{\max}=\ln\left[Rw_{\max}\sqrt{\gamma \omega^{2}}\right]$ is the intrinsic growth rate of a perfectly adapted population, with *P *=* *1 and$\overset{¯}{z}=\theta$. Equation (3.8) shows that strong frequency‐dependent selection is potentially devastating to the population. The maximum intrinsic growth rate (*r*_max_) determines the tolerable limit of the strength of frequency‐dependent selection; the population will inevitably decline to extinction when $P<e^{-r_{\max}}$. When $P>e^{-r_{\max}}$, population growth will be positive as long as the lag to the environmental optimum is not too large. Critical rates of environmental change (given $P>e^{-r_{\max}}$) are obtained by substituting Equation (3.6) into Equation (3.8) and solving for *b* (see Equation (2) of the main text).

The potential for extinction via asymmetric frequency‐dependent selection is not limited to contexts of environmental change. For example, Lande (1976) intuits that:

“As characters mediating dominance hierarchies and other forms of asymmetrical, frequency‐dependent selection are not uncommon, maladaptive evolution must be a fairly frequent event, and may play a significant role in some extinctions.”

In our model, extinction can occur within a constant environment (*b *=* *0) if frequency‐dependent selection drives the population far away from the environmental optimum. Population persistence requires that stabilizing selection (*γ*) exceeds the threshold defined by:

(3.9) $$\gamma_{\mathrm{crit}.}=\frac{e^{-2{\left[\mathrm{er}f^{-1}\left(2P-1\right)\right]}^{2}}}{\sigma^{2}\left(4\pi P^{2}\left(r_{\max}+\ln\left[P\right]\right)+e^{-2{\left[{\text{erf}}^{-1}\left(2P-1\right)\right]}^{2}}\right)}.$$

When *γ* < *γ*_crit_, the population is driven to extinction.

Through the second fitness component, the trait is subject to asymmetric *frequency‐dependent selection,* which favours directional change in the trait mean (see Lande, 1976). Asymmetric forms of FD selection can arise when competitive ability for resources or mates, or resistance to predation or parasitism, increases or decreases monotonically with trait expression (e.g., when trait size determines an individual's position within a dominance hierarchy; Lande, 1976), conditions that might be found under certain highly competitive sexual selection regimes that could result in elevated extinction risk (Doherty et al., 2003; Kokko & Brooks, 2003; Martins, Puckett, Lockwood, Swaddle, & Hunt, 2018). For simplicity, we arbitrarily assume that the frequency‐dependent component of selection leads to exclusion (truncation) of individuals at the lower range of the trait distribution from the breeding population, thereby favouring an increase in the trait mean (Figure 1). Our choice of the direction of FD selection is arbitrary, and the results of our model apply equally well in cases where FD selection acts against the upper tail of the trait distribution (i.e., favouring a decrease in the trait mean). While our model neglects symmetric forms of frequency dependence, which favour phenotypic extremes of the trait distribution without altering the trait mean (e.g., disruptive selection, or rare‐type advantage; Rueffler, Van Dooren, Leimar, & Abrams, 2006), we discuss potential effects of symmetric frequency dependence on evolutionary rescue further below.

In a stable environment, asymmetric FD selection is invariably maladaptive because it drives the population away from its environmental optimum (Box 3; see Lande, 1976 for discussion of a similar model). The deviation between the trait mean and the environmental optimum gives rise to opposing selection between the two fitness components, and at equilibrium, FD selection away from the optimum is offset by frequency‐independent selection towards it. Extinction is possible if the population's deviation from the optimum is large (e.g., when stabilizing selection is weak relative to FD selection; see Box 3).

In a changing environment, persistence depends on the population's ability to track the moving optimum. Following previous evolutionary rescue theory, we can define rates of environmental change that are tolerable and that will not drive the population extinct. In a population under frequency‐*independent* viability selection but lacking the frequency‐dependent selection component, the tolerable rate of environmental change falls within the range:

(1) $$-G\sqrt{2\gamma r_{\max}}<b<G\sqrt{2\gamma r_{\max}},$$

where *b* depicts the rate and direction of change of the environmental optimum, *G* is the additive genetic variance in the trait (i.e., the product of the trait's heritability, *h* ^2^, and its phenotypic variance, *σ* ^2^), *γ* is the strength of stabilizing selection towards the optimum, and *r*_max_ is intrinsic growth rate of a perfectly adapted population (see Box 3). Equation (1), which matches results from previous evolutionary rescue models (e.g., Chevin et al., 2010), illustrates that populations with high genetic variation, high reproductive capacity and strong selection are the least likely to go extinct.

When frequency‐dependent selection operates, there are two criteria for population persistence. First, the population must be able to tolerate the demographic cost of selection (a well‐known issue in animal breeding, similar to Haldane's cost of selection; see Haldane, 1957). Population fertility must be able to cope with the selective removal of individuals by way of FD selection. This condition is met as long as $P>e^{-r_{\max}}$, where *P* represents the proportion of individuals surviving early viability selection that then contributes to reproduction; 1 – *P* represents the fraction that is selectively eliminated by FD selection and provides a measure of the strength of FD selection. Second, the rate of environmental change (assuming $P>e^{-r_{\max}}$) must fall within the critical limits defined by:

(2) $$b_{\mathrm{crit}.}=\frac{G\sqrt{1-\gamma \sigma^{2}}}{P\sqrt{2\pi \sigma^{2}}}e^{-{\left[erf^{-1}\left(2P-1\right)\right]}^{2}}\pm G\sqrt{2\gamma \left(r_{\max}+\ln\left[P\right]\right)},$$

where *σ* ^2^ is the phenotypic variance of the trait, and erf^−1^(·) is the inverse error function (see Box 3). Note that the boundary limits in Equation (2) reduce to those in Equation (1) when there is no FD selection (i.e., when *P *=* *1).

**Figure 2:**
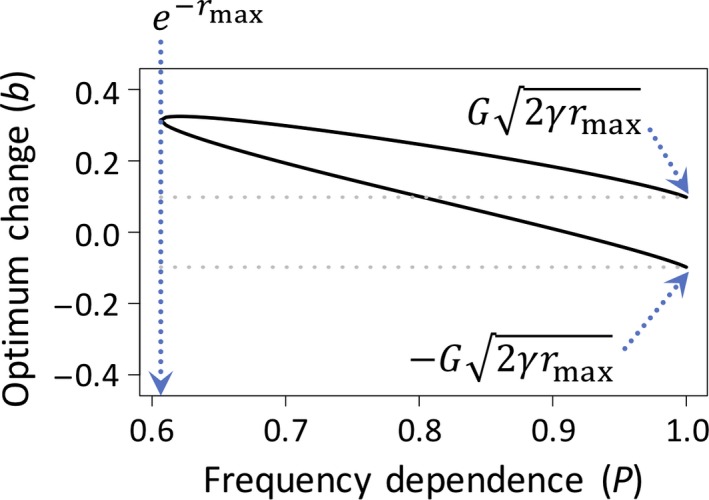
Frequency‐dependent selection and population persistence. The theoretical curves show the limits of a population's tolerance of environmental change as a function of the strength of frequency‐dependent selection. The region between the solid curves, which are based on Equation (2), shows the tolerable rates of change in the environmental optimum under frequency‐dependent selection (*P *<* *1). For values of *P* below the threshold, $e^{-r_{\max}}$, the population cannot persist under any circumstance; the threshold *P* corresponds to the point at which the top and bottom curves unite. For point of contrast, the area between the broken grey lines shows the tolerable rates of change in a population with no frequency‐dependent selection (*P *=* *1). All curves are based on Equation (2) with parameters *G *=* *0.5, *r*_max_ **=* *0.5, *ω* ^2^ **=* *25, *σ* ^2^ **=* *1, and *γ* = 1/(*ω* ^2^ + *σ* ^2^)

In Figure 2, we illustrate the effects of frequency‐dependent selection on population persistence. Three points stand out. First, FD selection decreases the potential for population persistence; with increasing intensity of FD selection (corresponding to decreasing *P*), the population tolerates an increasingly narrow range of environmental change. Second, frequency dependence creates a bias in the form of environmental change that is most (and least) likely to be tolerated by the population. Persistence is most likely when the environmental optimum shifts in the same direction as the orientation of the FD selection (*b *>* *0). This is because frequency dependence facilitates tracking of the environmental optimum when it shifts the population mean in the same direction in which the optimum changes. On the other hand, even small shifts of the optimum in the opposite direction (*b *<* *0) can have catastrophic consequences for population persistence, as there is little tolerance for change in that direction. Third, some conditions of FD selection and environmental change lead to evolutionary rescue of an otherwise doomed population. For example, a modest degree of FD selection can rescue a population at what would be the limit of tolerable environmental change in the absence of frequency dependence (i.e., weak FD selection allows the population to tolerate environmental change in excess of $b=G\sqrt{2\gamma r_{\max}}$).

### 2.2 Symmetric frequency‐dependent selection and evolutionary rescue?

Our model incorporates asymmetric FD selection, which modifies the net strength of directional selection on a trait, and thereby influences the potential for evolutionary rescue by helping or hindering the population's ability to track a moving environmental optimum (see above). However, other forms of FD selection may have different consequences for population persistence. Symmetric FD selection, including disruptive selection, does not alter the trait mean (Lande, 1976; Rueffler et al., 2006) and, therefore, will not directly affect the ability of the population to track the optimum. On the other hand, symmetric FD selection may indirectly affect evolutionary rescue by inflating the genetic variance of traits mediating adaptation to a novel environment. For example, disruptive selection prior to an abrupt change in environment may prime the population for rapid adaptation by maintaining an expanded pool of standing genetic variation in one or more key traits. This elevated genetic variance may increase the probability that rescue alleles with large fitness effects segregate within the population (Bell, 2017; Orr & Unckless, 2008, 2014), or it can reduce the time that adapting populations spend at critically small sizes where the risk of stochastic extinction is high (Gomulkiewicz & Holt, 1995; Gomulkiewicz & Houle, 2009). These verbal evolutionary arguments point to future opportunities for additional theoretical work to formalize how different scenarios of FD selection impact population persistence.

## 3 AN EMPIRICAL OVERVIEW OF THE LINKS BETWEEN FREQUENCY‐DEPENDENT SELECTION, GENETIC POLYMORPHISMS AND POPULATION MEAN FITNESS

Our quantitative genetic model (Box 3; Figure 2) was constructed to illustrate a biological situation where environment‐dependent and frequency‐independent selection first favours a phenotypic optimum that is subject to stabilizing selection, followed by a frequency‐dependent truncation selection episode, whereby individuals below or above a certain phenotypic threshold value are selectively removed from the breeding population (a form of asymmetric FD selection; Lande, 1976). How common and realistic are such biological situations? Actually, they might be quite common, as we argue below, particularly when selection changes sign during different parts of an organism's life cycle, which is a frequent biological scenario (Barrett, Rogers, & Schluter, 2008; Schluter, Price, & Rowe, 1991).

Consider, for instance, many sedentary breeding birds like hole‐breeding tits (Paridae), where a classical challenge is to time reproduction to a seasonally changing food supply (usually caterpillars) with a peak in late spring (Perrins, 1970). This seasonally ephemeral food peak constitutes an environmental optimum to which the birds are selected to synchronize the peak demand of their offspring (Charmantier et al., 2008; Chevin, Visser, & Tufto, 2015). Now, consider a later part in the life cycle, namely during territory establishment after the breeding season in early autumn of these sedentary birds. Obtaining a territory before winter is critical to survival and territories are in short supply, as the carrying capacity of the population is usually much lower than the number of offspring that are produced each year. As a result, populations of tits experience density‐dependent regulation (Lack, 1954), which intensifies territory competition among juveniles which try to establish themselves. During territory competition, the earliest hatched individuals are usually more successful in establishing themselves and obtain higher dominance status, due to an advantage of prior occupancy (Johansson, Smith, & Jonzen, 2014; Nilsson, 1989; Nilsson & Smith, 1988). As a consequence of density‐dependent population regulation and competition for a limited number of territories, a frequency‐dependent selection pressure to breed early relative to other individuals therefore arises. Such competition‐mediated frequency dependence can oppose the frequency‐independent selection pressure to breed later and synchronize nestling demands with the seasonal food peak (Kokko, 1999; Svensson, 1997; Svensson & Nilsson, 1995). A similar situation, albeit in reverse order, is experienced by long‐distance migratory birds that spend their winters in Africa, but which return to northern Europe for breeding each spring (Johansson & Jonzen, 2012a,b). Here, the frequency‐dependent selection episode happens before reproduction and the frequency‐independent selection process towards the environmental optimum, but again it is important to arrive early to the breeding grounds, before competitors (Kokko, 1999; Johansson & Jonzen, 2012a,b). The result in both cases is a tension between selection for being earlier than your competitors (a frequency‐dependent process) and selection to match the environmental food peak (a frequency‐independent process), which has been modelled using game theoretical approaches (Kokko, 1999; Johansson & Jonzen, 2012a,b; Johansson et al., 2014).

There are other biologically realistic situations with similar tensions between frequency‐independent and frequency‐independent selection episodes and fitness components. Some of these are seen in sexual selection and competition for mates, rather than for food resources or survivorship. One general situation is protandry: the emergence or arrival of males before females on breeding grounds (Morbey & Ydenberg, 2001). The selection pressures driving protandry have been discussed at length, and there are many different hypotheses for why it evolves (Morbey & Ydenberg, 2001). One explanation is based on the frequency‐dependent advantages (priority benefits) of early emergence over limited territories that are needed to successfully attract females (Kokko, 1999). Again, it is important to arrive early relative to competitors, rather than early in an absolute sense, and such competition‐driven processes can create a mismatch between the environmental optimum that should maximize population growth rates and the frequency‐dependent selection on individuals (Kokko, 1999). For instance, in many species of butterflies and other insects in temperate regions, males typically emerge earlier in spring or summers than females and this is usually interpreted as a result of intrasexual competition for mating opportunities (Fagerstrom & Wiklund, 1982; Svensson & Waller, 2013; Wiklund & Fagerstrom, 1977).

Our final empirical example of the relationship between frequency‐dependent selection and population fitness comes from sexual conflict research. The relationship between individual (relative) fitness and absolute (mean) population fitness has recently gained increased attention in this field (Berger et al., 2016; Rankin et al., 2011). This increased interest and realization that one needs to distinguish between individual relative fitness and absolute mean population fitness in sexual conflict research have a clear parallel to the evolutionary rescue literature, where this distinction is also crucial if one wishes to understand both genetic and ecological dynamics, including extinction risk (Gomulkiewicz & Holt, 1995). Specifically, traits that are favoured in males might not necessarily maximize population mean fitness, whereas the opposite might be true for traits favoured in females (Berger et al., 2016), since population growth is more tightly coupled to female than to male fitness components (Harts, Schwanz, & Kokko, 2014). For instance, sexual selection on males can favour aggressive genotypes with high mean relative fitness, even if such genotypes depress population growth rate by reducing female fecundity, resulting in a “Tragedy of the Commons” (Berger et al., 2016; Rankin et al., 2011). Moreover, traits that improve a male's ability to compete for mating opportunities can also be negatively genetically correlated with traits associated with female reproductive success (Chippindale, Gibson, & Rice, 2001; Poissant, Wilson, & Coltman, 2010). Correlated evolutionary responses of females to selection in males can therefore potentially hinder female adaptation and reduce population productivity (Connallon, Cox, & Calsbeek, 2010; Kokko & Brooks, 2003; Lande, 1980).

**Figure 3:**
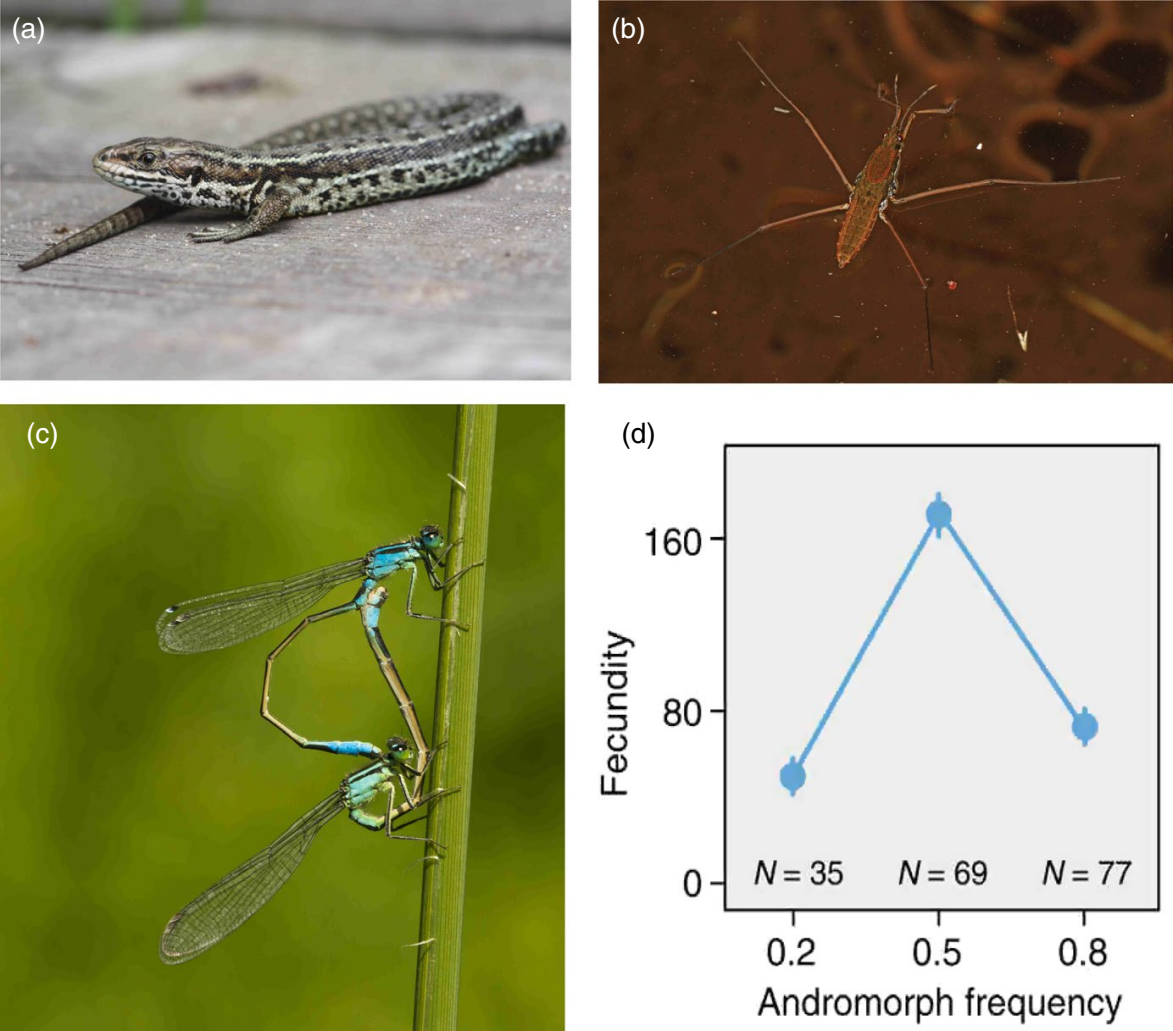
Empirical examples of studies that link sexual conflict and frequency‐dependent selection to mean population fitness. (a) In common lizards (*Zootoca vivipara*), male‐biased sex ratios in field enclosures lead to sexual conflict and population collapse, due to reduced female fitness and thereby reduced population fitness (photograph by Alastair Rae/Wikimedia Commons). (b) Similarly, in water striders (*Aquarias remigis*) sexual conflict favours aggressive males within populations which harass females and thereby depress population mean fitness, whereas the spread of such aggressive male phenotypes is opposed by higher‐level selection and presumably higher extinction risk of populations with a high frequency of aggressive males (photograph by Judy Gallagher/Wikimedia Commons). (c**)** In the common bluetail damselfly (*Ischnura elegans*), there are three heritable female colour morphs, one which is male coloured (“male mimics”), and these three morphs are maintained by frequency‐dependent sexual conflict (Le Rouzic et al., 2015; Svensson et al., 2005). Here is a male mating with a male mimic (Photograph by Erik Svensson). (d) Experimental manipulations of the frequency of the male mimic in field enclosures reveal that average female fecundity (closely connected to population mean fitness) is maximized at intermediate morph frequencies, providing a link between sexual conflict, genetic variation, frequency‐dependent selection and population mean fitness (Takahashi et al., 2014)

Although there is little empirical evidence for the above scenarios of sexual conflict from natural (field‐based) systems, they have received empirical support from laboratory studies in seed beetles (Berger et al., 2016) and from experimental mesocosm studies in common lizards (*Zootoca vivipara*), water striders (*Aquarius remigis*) and damselflies (*Ischnura elegans*; Figure 3). For example, in common lizards, experimental manipulations of sex ratios resulted in lower female fitness and hence increased extinction risk, when sex ratios were male‐biased, demonstrating a clear link between sexual conflict through male mating harassment, female fitness and population mean fitness (Le Galliard, Fitze, Ferriere, & Clobert, 2005). In water striders, sexual conflict through male mating harassment of females is common and can decrease female fitness and favour increased dispersal (Eldakar, Dlugos, Pepper, & Wilson, 2009; Eldakar, Dlugos, Wilcox, & Wilson, 2009). Whereas aggressive males that harass females to obtain matings are favoured within populations when dispersal is limited, populations containing many such harassing male phenotypes might show higher extinction risk, resulting in a conflict between individual‐level selection and higher‐level selection at the level of demes (Eldakar, Dlugos, Pepper et al., 2009; Eldakar, Dlugos, Wilcox et al., 2009).

The studies mentioned above establish links between sexual conflict, individual‐level frequency‐dependent selection, population fitness and (potentially) to multi‐level selection through the Tragedy of the Commons (Le Galliard et al., 2005; Eldakar, Dlugos, Wilcox et al., 2009). These studies also show how frequency‐dependent selection can reduce population mean fitness, through frequency‐dependent selection on males. However, missing from both studies is a genetic component for the traits responsive to selection and the possibility that frequency‐dependent selection can operate on females and counteract male mating harassment, potentially mitigating the effects of males on population mean fitness. As such an example, consider the situation in damselflies, where sex‐limited female colour polymorphisms are common (Svensson, Abbott, Gosden, & Coreau, 2009). One female colour morph is typically a “male mimic” with a clear function in sexual conflict avoidance (Neff & Svensson, 2013; Svensson et al., 2005, 2009). Such female colour polymorphisms are maintained by frequency‐ and density‐dependent sexual conflict, mediated by male mating harassment directed towards common morphs (Gosden & Svensson, 2009; Le Rouzic, Hansen, Gosden, & Svensson, 2015; Figure 3d). Experimental manipulations of female morph frequencies in three different treatments (20%, 50% and 80% of the male mimic) revealed that population fitness (mean female fecundity) was maximized when morph frequencies were unbiased and approximately equal (Takahashi, Kagawa, Svensson, & Kawata, 2014), demonstrating a link between frequency‐dependent selection, genetic polymorphisms and population mean fitness (Svensson, 2017). Interestingly, there are no monomorphic populations documented for this study species (Gosden, Stoks, & Svensson, 2011), implying that frequency‐dependent selection at the individual level and elevated extinction rates of populations with biased morph frequencies (and hence lowered population mean fitness) might both contribute to maintain this polymorphism at the continental scale (Svensson, 2017; Takahashi et al., 2014). These studies also suggest a potential link between evolutionary rescue and sexual conflict. More specifically, females can evolve fitness tolerance or resistance to male mating harassment and in some species even sex‐limited female polymorphisms (Gosden & Svensson, 2009; Karlsson, Kovalev, Svensson, & Gorb, 2013; Karlsson, Svensson, Bergsten, Hardling, & Hansson, 2014; Le Rouzic et al., 2015; Neff & Svensson, 2013; Ronn, Katvala, & Arnqvist, 2007; Svensson & Raberg, 2010; Svensson et al., 2005; Takahashi et al., 2014). Once such defensive female traits or polymorphisms have evolved, they might counteract the negative impact of sexual conflict on population mean fitness. Various forms of female defence traits thus represent a form of evolutionary rescue from sexual conflict (Gomulkiewicz & Holt, 1995). Therefore, the likelihood of population extinction due to sexual conflict (Rankin et al., 2011) might be reduced by evolutionary rescue restoring female fitness and the coevolution of female resistance or female fitness tolerance to male mating harassment (Gosden & Svensson, 2009; Ronn et al., 2007; Svensson & Raberg, 2010). Other forms of frequency‐dependent selection associated with sexual conflict or highly competitive forms of sexual selection might, however, be associated with elevated extinction risk (Doherty et al., 2003; Kokko & Brooks, 2003). For instance, a recent study on fossil ostracods revealed elevated extinction risk associated with high degree of sexual size and shape dimorphism (Martins et al., 2018), and such outcomes are also predicted in models of asymmetric FD selection when relative, rather than absolute trait values could push a species or population from its environmental optimum (Lande, 1976).

## 4 CONCLUSIONS

Here, we have discussed the rich theoretical literature and the more limited empirical literature that links frequency‐dependent selection to population mean fitness and its evolutionary consequences. We have further explored the relationship between frequency‐dependent selection and population mean fitness when the environment changes in a simple quantitative genetic model, and the implications for evolutionary rescue. We find that the relationship between frequency‐dependent selection and population mean fitness is complex and will vary depending on the demographic, ecological and genetic details of the population or species. These complications aside, the different scenarios are sufficiently interesting to merit further theoretical and empirical work, and our hope with this article is that we have provided a foundation for such research. Frequency‐dependent selection can either facilitate evolutionary rescue and increase population mean fitness or decrease it (Figure 2). Therefore, frequency‐dependent selection might be much more important to ecological and evolutionary dynamics of populations than might have been realized in the past and its importance goes beyond its traditionally recognized role in maintaining genetic polymorphisms within populations (Ayala & Campbell, 1974).

Our findings are also relevant to the classical question of how selection and genetic variation affect population fitness (Haldane, 1937, 1957; Wallace, 1970, 1975), and whether genetic polymorphisms will reduce extinction risks as argued by some (Forsman, 2016) or increase extinction risks as argued by others (Bolton, Rollins, & Griffith, 2015). Answering these questions requires that one considers genetic and ecological details which are central to the problem. This is one context where explicit population and quantitative genetic models, with demographic feedbacks might provide more insights than game theory and adaptive dynamics models (Boxes 1–3; Svensson, 2017; Lion, 2018). Finally, frequency dependence and other non‐random processes can also shape diversity above the population or species level, such as ecological communities, and can thereby maintain both species diversity and genetic diversity (Ayala, 1971; Harpole & Suding, 2007; Svensson et al., 2018; Wills et al., 2006)**.** The potentially complex relationships between phenotypic selection, genetic diversity and higher‐level processes of population fitness, extinction risk and the productivity of populations or communities also have interesting applied consequences. For example, the conflict between lower‐level processes among individual genotypes and higher‐level effects in terms of population performance and productivity are worth consideration in attempts to improve crop yield in evolutionary agroecology, for example, by experimentally altering the balance between lower‐ and higher‐level processes using multi‐level selection theory (Weiner et al., 2010). Whether FD selection improves population mean fitness or decreases it and thereby causes maladaptation is ultimately an empirical question that should be addressed in future studies. The theoretical exploration we have performed in this paper suggest that FD selection can either increase or decrease maladaptation, depending on whether it is aligned with the direction of frequency‐independent selection caused by abiotic factors.
